# TDP-43 Pathology in Alzheimer’s Disease

**DOI:** 10.1186/s13024-021-00503-x

**Published:** 2021-12-20

**Authors:** Axel Meneses, Shunsuke Koga, Justin O’Leary, Dennis W. Dickson, Guojun Bu, Na Zhao

**Affiliations:** Department of Neuroscience, Mayo Clinic, 4500 San Pablo Road, Jacksonville, FL 32224 USA

**Keywords:** Alzheimer’s disease, TDP-43, *TARDBP*

## Abstract

Transactive response DNA binding protein of 43 kDa (TDP-43) is an intranuclear protein encoded by the *TARDBP* gene that is involved in RNA splicing, trafficking, stabilization, and thus, the regulation of gene expression. Cytoplasmic inclusion bodies containing phosphorylated and truncated forms of TDP-43 are hallmarks of amyotrophic lateral sclerosis (ALS) and a subset of frontotemporal lobar degeneration (FTLD). Additionally, TDP-43 inclusions have been found in up to 57% of Alzheimer’s disease (AD) cases, most often in a limbic distribution, with or without hippocampal sclerosis. In some cases, TDP-43 deposits are also found in neurons with neurofibrillary tangles. AD patients with TDP-43 pathology have increased severity of cognitive impairment compared to those without TDP-43 pathology. Furthermore, the most common genetic risk factor for AD, apolipoprotein E4 (*APOE4*), is associated with increased frequency of TDP-43 pathology. These findings provide strong evidence that TDP-43 pathology is an integral part of multiple neurodegenerative conditions, including AD. Here, we review the biology and pathobiology of TDP-43 with a focus on its role in AD. We emphasize the need for studies on the mechanisms that lead to TDP-43 pathology, especially in the setting of age-related disorders such as AD.

## Background

Alzheimer’s disease (AD), the leading cause of dementia, is a heterogeneous neurodegenerative disorder in terms of clinical presentations and the density and distribution of the cardinal neuropathologic lesions. The neuropathologic hallmarks of AD are senile plaques composed of extracellular deposits of amyloid-β (Aβ) and neurofibrillary tangles composed of intracellular aggregates of tau protein with multiple post-translational modifications including phosphorylation. Senile plaques are complicated and heterogeneous lesions that contain not only amyloid deposits and tau positive neurites, but also neurites with degenerating pre- and post-synaptic elements (so-called dystrophic neurites), as well as activated microglia and reactive astrocytes [1]. Aβ deposits within the walls of blood vessels in the form of amyloid angiopathy are found in many patients with AD, but it is also found in other neurologic disorders [2]. Tau deposits are also found in neuronal cell processes (“neuropil threads”) and in dystrophic neurites within senile plaques [1, 3]. Neurofibrillary tangles are not exclusive to AD, but are found in a wide range of neurological disorders [4], as hereditary disorders [5] or secondary pathologic processes [6], due to environmental or genetic factors. Based on the density of neurofibrillary tangles in the hippocampus relative to those in the neocortex, AD can be classified into three clinicopathologic subtypes: typical AD, hippocampal sparing AD, and limbic predominant AD [7]. The clinicopathologic classification of AD subtypes has recently been confirmed and extended in living patients with neuroimaging methods [8, 9], identifying additional subtypes, including minimal change AD and AD with asymmetrical neocortical involvement.

Clinically, the two major presentations of AD can be classified as amnestic and non-amnestic. The former is characterized by deficiencies in short-term memory, recall and learning, which are the most common clinical presentations of typical and limbic predominant subtypes of AD. The latter shows impairment in other cognitive domains, such as language, visuospatial skills, or executive functioning. This is often associated with hippocampal sparing AD.

In addition to senile plaques and neurofibrillary tangles, many AD brains have other pathological lesions, such as cerebrovascular pathology, Lewy bodies, argyrophilic grain disease, hippocampal sclerosis, cerebral amyloid angiopathy, and transactive response DNA binding protein of 43 kDa (TDP-43) pathology [10, 11]. Importantly, these additional pathologies significantly increase the risk for dementia compared to patients with only one pathology [12]. The mixed pathologies also lower the threshold and accelerate the progression for clinical diagnosis of AD [13]. More recently, Spina and coworkers systematically investigated co-pathologies in early-onset and late-onset AD patients and found that the number of co-pathologies was associated with worse cognitive performance [11]. In this review, we focus on TDP-43 in aging and AD from clinical, pathological, and basic research perspectives.

### Biology of TDP-43

TDP-43 is a 43 kDa heterogeneous nuclear ribonuclear protein (hnRNP) composed of 414 amino acids and is encoded by the *TARDBP* gene located on chromosome 1 (1p36.22) [14]. TDP-43 is synthesized in the cytoplasm and shuttled into the nucleus where it primarily resides to perform its physiological functions.

#### Biological function of TDP-43

The function of TDP-43, much like other hnRNPs, is to regulate gene expression and other aspects of RNA processing including RNA splicing, mRNA turnover, RNA trafficking, and microRNA (miRNA) biogenesis [15–22]. TDP-43 targets over 4,000 different mRNA transcripts [23], ranging from disease-associated transcripts [18], to its own mRNA transcript [17]. Disruption of the proper regulation of TDP-43 may contribute to its pathogenesis. Studies have shown that TDP-43 self-regulates through a negative feedback loop where TDP-43 destabilizes its mRNA transcript by binding to the 3’ untranslated region [17]. Interestingly, TDP-43 has been shown to down-regulate tau expression by destabilizing its mRNA transcripts [18]. Furthermore, TDP-43 might regulate the ratio of 4-repeat tau and 3-repeat tau via alternative splicing of tau exon 10 [24]. However, the regulation of tau expression by TDP-43 was not replicated in another independent study of AD [25]. Thus, the relationship between TDP-43 and the expression of tau remains unclear and needs to be further investigated.

Additionally, TDP-43 plays a role in the cellular stress response [15, 26–28]. If a cell is exposed to certain stressors (i.e., heat shock, oxidative stress, or viral infection), it can regulate levels of mRNA to conserve energy and prioritize cell survival [29, 30]. Stress granules are cytoplasmic foci in response to cellular stress that contain non-essential RNA. TDP-43 associates with ribosomes in stress granules to temporarily halt translation and promote cytoprotective protein synthesis [15, 31].

TDP-43 has been reported to regularly shuttle between the cytoplasm and nucleus depending on transcriptional needs [32]. Interestingly, low levels of TDP-43 have even been found to reside inside of mitochondria in human motor and cortical neurons; however, age-matched neurons from amyotrophic lateral sclerosis (ALS) and frontotemporal lobar degeneration (FTLD) patients expressed a significantly higher amount of mitochondrial TDP-43, reportedly altering their morphology and impairing mitochondrial function [33].

#### Protein structure of TDP-43

**Fig. 1:**
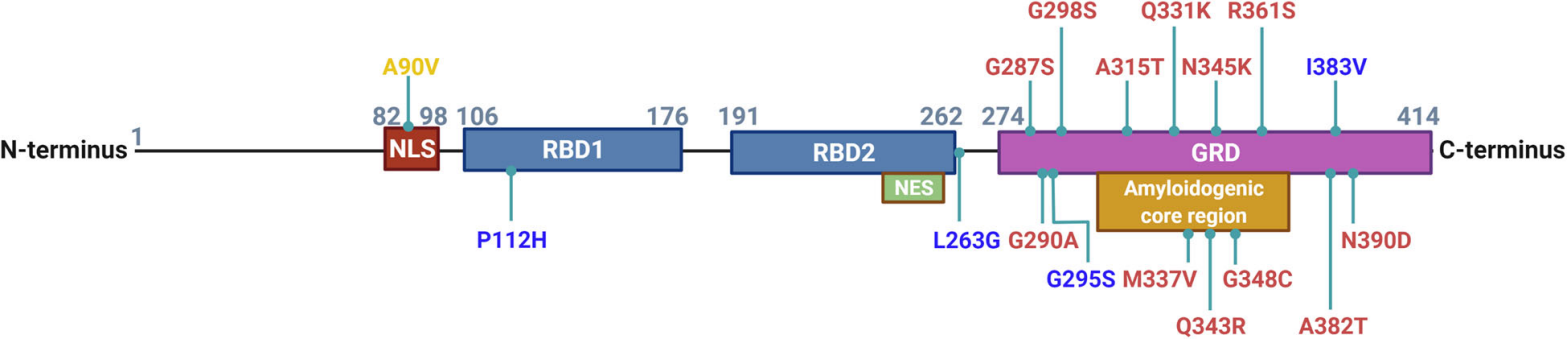
Protein structure of transactive response DNA binding protein of 43 kDa (TDP-43). TDP-43 is a 414 amino acid protein with a nuclear localization sequence (NLS) followed by two RNA binding domains (RBD1 and RBD2), a nuclear export sequence (NES), and a glycine rich prion-like (GRD) C-terminus. The mutations reported to increase the risk of ALS (red), FTLD (blue), and AD (orange) are indicated

The structure of TDP-43 is composed of an N-terminal domain, a nuclear localization sequence (NLS), two RNA binding domains (RBD1 and RBD2), a nuclear export signal (NES), and a C-terminal glycine rich domain (GRD) (Fig 1) [34]. The protein also has an amyloidogenic core region (residues 311-360) with two alpha-helices that convert into beta sheets in TDP-43 aggregates [35]. The NLS is critical for physiological function, as mutations or deletions of the NLS result in mislocalization and aggregation of TDP-43 that are characteristic of disease models [36–38]. Importin-α facilitates the transport of TDP-43 into the nucleus by binding to the NLS. The role of the NES in TDP-43 remains controversial. The export of TDP-43 from nucleus to cytoplasm is thought to be mediated by exportin XPO1 binding to the NES in the second RBD [39, 40]; however, recent data suggests that the export of TDP-43 from the nucleus does not require either XPO1 or the NES, but instead is exported through passive diffusion [37, 41–44]. The function of the N-terminus is to regulate the homodimerization of TDP-43 to ensure proper folding and mRNA splicing [45]. The C-terminus is important for mRNA splicing and hnRNP interactions, and it is also thought to play a role in the formation of TDP-43 inclusions [46]. Additionally, this portion of the protein has been referred to as a prion-like domain due to its low complexity and high proclivity for aggregation, as well as being the site for over 50 sporadic and familial ALS-associated mutations [34, 47–49].

#### Polymorphisms of *TARDBP* gene and disease risks

Mutations in the *TARDBP* gene are mainly associated with ALS and located along the glycine rich C-terminal domain (Fig 1). In particular, *TARDBP* mutants, such as Q331K and M337V, have been well studied for their associations to ALS [50]. Fewer *TARDBP* mutations, including P112H [51] and G295S [52], have been linked to FTLD. A reported mutant, I383V, has been implicated in both ALS and FTLD [52–55]. In general, most of the disease-associated mutations in the *TARDBP* gene are associated with an increase in TDP-43 aggregation and toxicity [48]. Interestingly, there are reports of a rare missense mutation in the NLS region of TDP-43, A90V, which is speculated to increase the risk of AD through a loss-of-function mechanism [56–58].

### TDP-43 pathology in ALS and FTLD

Pathological forms of TDP-43 were first identified in 2006 when ALS and FTLD patients were found to have tau-negative, ubiquitin-positive cytoplasmic inclusion bodies [59–61]. The pathogenic mechanisms in these brains ultimately result in TDP-43 depletion from the nucleus, TDP-43 mislocalization into the cytoplasm, and the formation of insoluble aggregates that contain TDP-43 with multiple posttranslational modifications including ubiquitination, phosphorylation, and truncation [59–63]. These TDP-43 inclusion bodies found in neurons, neuronal cell processes, and glia are now characteristic of the pathology in the most common forms of ALS and FTLD [60, 63, 64].

#### Subtypes of TDP-43 pathology in ALS and FTLD

**Fig. 2:**
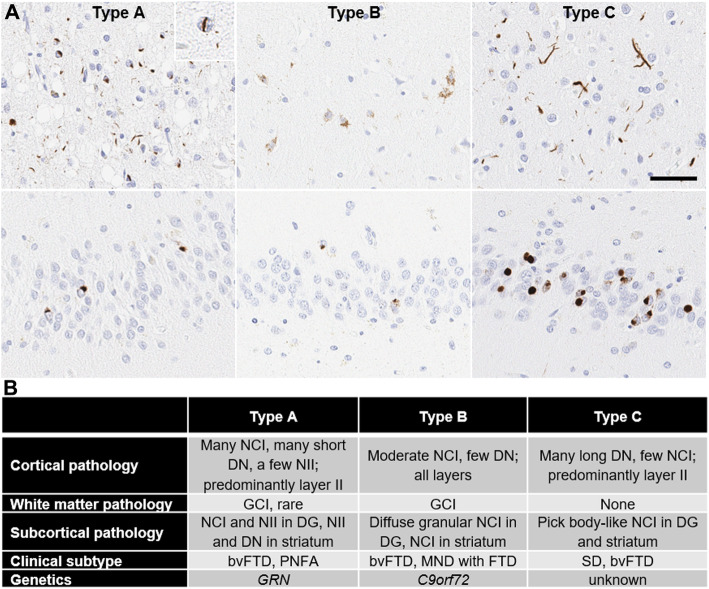
Representative images of TDP-43 pathology subtypes in FTLD-TDP brains. (A) Immunohistochemistry with an anti-phosphorylated-TDP-43 antibody (pSer409/pSer410) shows numerous neuronal cytoplasmic inclusions, short dystrophic neurites, and neuronal intranuclear inclusion (inset) in Type A; diffuse granular neuronal cytoplasmic inclusions in Type B; and numerous thick and long dystrophic neurites in Type C, in the superficial layer of the midfrontal gyrus (upper panel). In the dentate gyrus (lower panel), Type A shows compact neuronal cytoplasmic inclusions; Type B shows diffuse granular neuronal cytoplasmic inclusions; and Type C shows Pick body-like neuronal cytoplasmic inclusion. Scale bar = 50 μm. (B) A summary of clinical, pathological, and genetic features of TDP-43 pathology subtypes. NCI: Neuronal cytoplasmic inclusion; NII: Neuronal intranuclear inclusion; DN: Dystrophic neurite; GCI: Glia cytoplasmic inclusion; DG: Dentate gyrus; FTD: Frontotemporal dementia; bvFTD: Behavioral variant frontotemporal dementia; PNFA: Progressive non-fluent aphasia; MND: Motor neuron disease; and SD: Semantic dementia

Based on the morphology, cell type, and distribution of TDP-43 pathology, FTLD-TDP can be classified into four main subtypes [65–69] (Fig 2). Type A is characterized by compact neuronal cytoplasmic inclusions (NCIs) and short dystrophic neurites (DNs) with occasional neuronal intranuclear inclusions (“cat-eye” inclusions) (NIIs) distributed preferentially in upper neocortical layers. Type B is characterized by diffuse granular NCIs and sparse DNs with inclusions showing no preference for superficial or deep neocortical layers. Oligodendroglial cytoplasmic inclusions are common in affected cortices and subcortical white matter, especially Type B cases associated with motor neuron disease. Type C is characterized by numerous DNs predominantly in superficial and deep neocortical layers, which are longer and thicker than those seen in Type A. Sparse NCIs are detected in the neocortex, but dense, compact, and round NCIs (“Pick body-like”) are frequent in the hippocampal dentate gyrus and in the basal ganglia, especially the putamen. The most distinctive feature of Type D is the presence of numerous NIIs, including both round inclusions and “cat-eye” type inclusions. Type D has variable DNs and NCIs. A fifth subtype, Type E, has been proposed [68], but it is less widely accepted. The characteristic features of Type E are granulofilamentous neuronal inclusions, abundant grains, and oligodendroglial inclusions that affect all layers of the neocortex. Among the TDP-43 subtypes, Type A is the most common type, followed by Type B. This pathologic subtyping has a good correlation with clinical phenotypes and genetics. Type A is most often associated with behavioral variant frontotemporal dementia (bvFTD) or progressive non-fluent aphasia (PNFA), while Type B is associated with bvFTD with or without motor neuron disease (MND). Most cases of FTLD due to *GRN* mutations have Type A; many, but not all cases of FTLD with *C9ORF72* mutations have Type B. Semantic dementia (SD) and bvFTD are common clinical phenotypes in Type C, but no genetic association has been reported. Type D is associated with frontotemporal dementia (FTD) and Paget’s disease of bone caused by mutations of *VCP* gene [70]. This classification has been demonstrated to be supported by clinical, biochemical, and genetic correlational studies for FTLD-TDP [69], but needs to be further examined in AD cases to evaluate its pathological significance.

#### Progression pattern of TDP-43 pathology in ALS and FTLD

Pathological progression of TDP-43 varies depending upon the underlying neurodegenerative disease with different progression patterns proposed for FTLD and ALS by Brettschneider et al [71, 72]. In bvFTD, stage 1 is associated with the lowest level of TDP-43 pathology in the basal and anterior portions of the prefrontal cortex, the pathology then invades other regions of the prefrontal cortex including the middle frontal gyrus and insular cortex as stage 2, leading into the motor cortex and parietal cortical areas as stage 3, and finally  reaches stage 4, the most advanced stage, with widespread and high density TDP-43 pathology involved in the occipital cortex [71]. The staging scheme for ALS includes early involvement of the motor cortex, brainstem and spinal cord (stage 1), prefrontal cortex (stage 2), postcentral cortex and striatum (stage 3),  and finally,  TDP-43 pathology infiltrates the anteromedial temporal lobe (stage 4)  [72].

#### Pathogenesis of TDP-43 in ALS and FTLD

Ubiquitination, phosphorylation, and truncation modifies the conformation of TDP-43, as well as its size and charge, contributing to the decreased shuttling into the nucleus [73–75]. Ubiquitin commonly binds to proteins to target them for eventual degradation. Lys-84, one of the multiple TDP-43 ubiquitination sites, is reported to be involved in the nuclear import of TDP-43 [76]. TDP-43 is phosphorylated most often at serine residues but can also be phosphorylated at tyrosine or threonine residues. The serine residues most often affected are serines 403, 404, 409 and 410; with serines 409 and 410 being the most common [77, 78]. Cytoplasmic TDP-43 can be cleaved by calpains and caspases into N-terminal fragments and C-terminal fragments (CTFs) with molecular weights of 35 and 20-25 kDa, respectively [79–81]. These fragments, in particular the CTFs, have been found to induce formation of ubiquitinated and phosphorylated cytoplasmic TDP-43 aggregates *in vitro* [82]. It is possible that neither phosphorylation nor ubiquitination is necessary for TDP-43 aggregation. Early-stage inclusions are neither ubiquitinated nor phosphorylated, and ubiquitination is usually associated with late stages in the aggregation process of *in vitro* neuronal cell culture models [79, 83]. Additionally, the potential lack of the NLS, precluding TDP-43 from shuttling back to the nucleus, may contribute to formation of aggregates [73].

An impairment in the clearance of TDP-43 may also contribute to the pathogenic process. An *in vitro* study has indicated that soluble TDP-43 is degraded by the ubiquitin-proteasome system, while insoluble TDP-43 aggregates are degraded via the autophagy system [84]. Other investigators determined that TDP-43 has a KFERQ-like sequence in the RBD1 domain, specifically QVKKD (amino acids 134 to 138), that allows Hsc70 binding and degradation of soluble, non-aggregated TDP-43 by chaperone-mediated autophagy [85]. Interestingly, degradation of TDP-43 species, particularly the CTFs, were much higher by the ubiquitin-proteasome system than by autophagy [85], suggesting that TDP-43 can be cleared through both mechanisms depending on its specific form.

In a non-diseased state, a balance between soluble and insoluble forms of RNA binding proteins (including TDP-43) and cell stress granules is maintained in the cytoplasm primarily due to their reversibility during cellular stress response [31]. In ALS and FTLD, this balance is possibly compromised due to the increased presence of aggregated TDP-43 within the cytoplasm, which in turn may increase cellular stress that leads to the formation of  additional stress granules and the  aggregation of RNA binding proteins, acting as seeds for TDP-43 aggregation [31]. TDP-43 can also be found within stress granules themselves depending on the conditions used to induced stress. For example, stress induced by sodium arsenite produces increased TDP-43 in stress granules [15, 86]. It has also been reported that TDP-43 inclusion bodies co-localize with markers of stress granules [26, 78, 86–89]. Interestingly, only the full length TDP-43 species, but not the CTFs, are recruited into stress granules, which requires both the RBD1 and GRD domains [90]. On the other hand, some investigators suggested that co-localization of TDP-43 with stress granules depends on RNA-bound forms of TDP-43. RNA-bound TDP-43 in stress granules is soluble, while free TDP-43 can form insoluble aggregates independent of stress granules [15, 91]. Together, the relationship between stress granules and TDP-43 pathology is a research focus that needs further investigation.

#### Gain of toxic and loss of normal function of TDP-43 in ALS and FTLD

Neuronal death associated with pathological TDP-43 is thought to be caused by a combination of both a toxic gain of function, as well as a loss of physiological function associated with depletion of TDP-43 from the nucleus [73]. Oligomeric and cytoplasmic aggregates of TDP-43 have been shown to be cytotoxic both *in vitro* and *in vivo* [92–95]. Additionally, mislocalized and aggregated TDP-43 can enhance mislocalization of nuclear TDP-43 and hinder intracellular transport [20, 47, 96–98]. Cytoplasmic mislocalization of TDP-43 may predispose the cell to stress since this has been shown to be associated with markers of stress response in cell culture model systems [47, 91, 99, 100].

Loss of function of TDP-43 is another mechanism implicated in neuronal loss in ALS and FTLD. Studies in mouse models rarely detect TDP-43 cytoplasmic inclusion bodies; however, neurodegeneration associated with loss nuclear TDP-43 can be evident [101]. In humans with *C9ORF72*-linked FTLD, there is loss of nuclear TDP-43 at pre-symptomatic stages [102]. Furthermore, the mere lack of nuclear TDP-43 is sufficient to cause neuronal atrophy [103]. This observation suggests that loss of nuclear TDP-43 is an early pathological event that might drive neurodegeneration. Additionally, loss of nuclear TDP-43 may modify chromatin accessibility leading to altered gene expression [20, 27, 47, 97, 104–111].

Interestingly, nuclear TDP-43 suppresses splicing of non-conserved cryptic exons, reducing the number of frameshift or nonsense mutations in mRNA transcripts [104, 112]. Patients with ALS or FTLD have impairments in non-conserved cryptic exon suppression function leading to the decay of mutated transcripts and disturbance in translation [110, 111, 113]. Cryptic exon splicing has also been noted in AD with TDP-43 pathology, including those with cytoplasmic inclusion bodies and those with only nuclear depletion of TDP-43, suggesting that impairments of TDP-43 cryptic exon repression may be an early event in TDP-43 pathogenesis in FTLD, ALS and a subset of patients with AD [114].

### TDP-43 pathology in AD

**Fig. 3:**
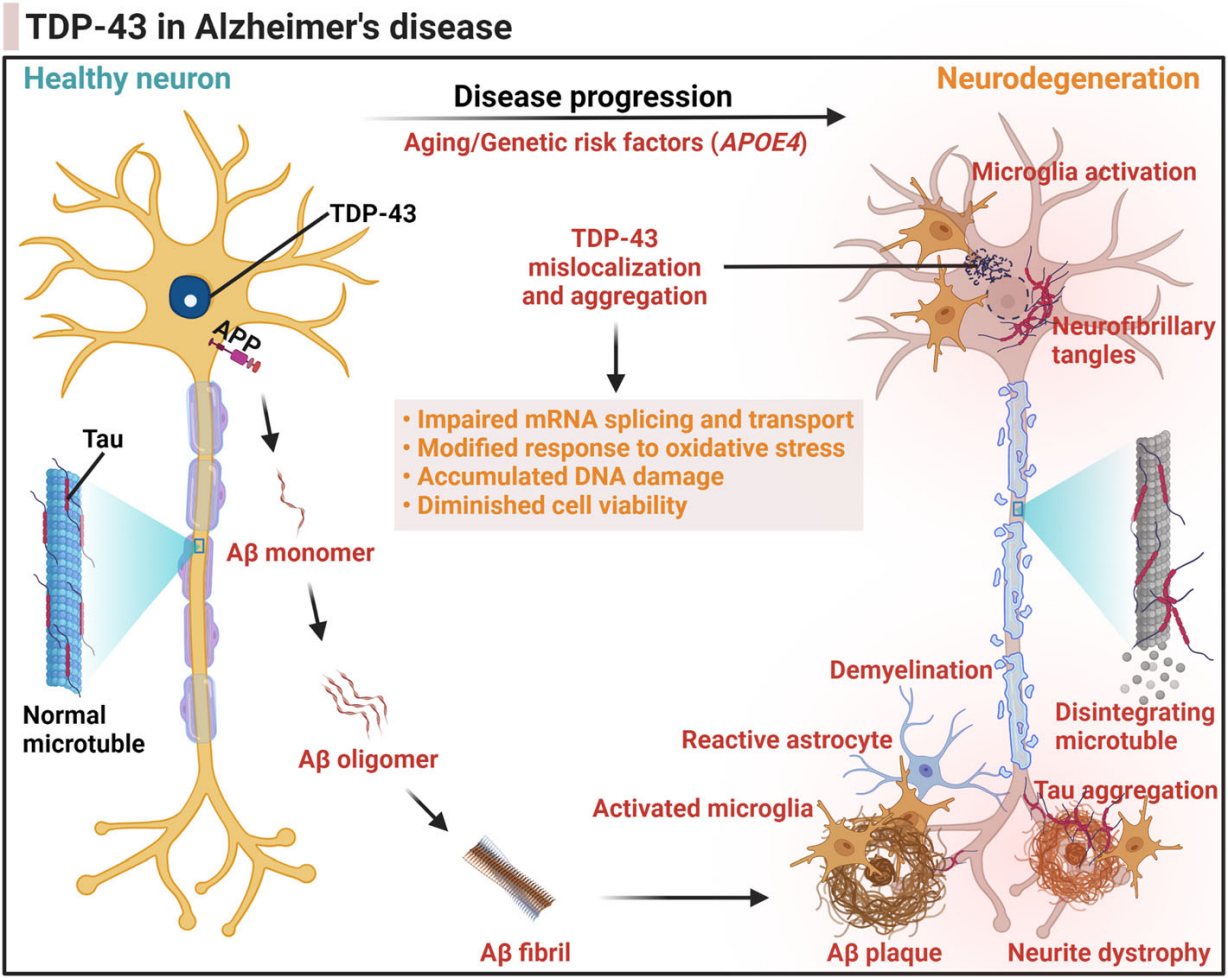
Illustration of the involvement of TDP-43 in the progression of Alzheimer’s disease. In the brain of Alzheimer’s disease (AD), the amyloid-β (Aβ) peptide is produced through the proteolytic processing of a transmembrane protein, amyloid precursor protein (APP) by β- and γ-secretases. The accumulation of soluble Aβ monomers in the brain parenchyma leads to the formation of Aβ oligomers, fibrils, and eventually Aβ plaques, due to overproduction and/or impaired Aβ clearance pathways contributing to the development of AD. The pathological changes of tau protein decrease its microtubule binding capacity and disrupts microtubule stability causing microtubule disintegration. The intracellular aggregates of tau protein form the neurofibrillary tangles. Tau deposits are also found in neuronal cell processes (“neuropil threads”) and in dystrophic neurites within Aβ plaques. Aβ plaques are heterogeneous lesions containing not only amyloid deposits and tau-positive neurites, but also neurites with degenerating pre- and post-synaptic elements (neurite dystrophy), as well as activated microglia, reactive astrocytes, and dysfunction of oligodendrocytes causing demyelination. TDP-43 is synthesized in the cytoplasm and retains the ability to shuttle from the cytoplasm into the nucleus where it primarily resides to perform its physiological functions such as RNA splicing. During the progression of AD, the pathogenic events lead to TDP-43 depletion from the nucleus, TDP-43 mislocalization into the cytoplasm, and the formation of insoluble TDP-43 aggregates. The neurodegeneration brought about by pathological TDP-43 can be caused by a potential combination of both a loss of physiological function and a gain of toxic functions

TDP-43 pathology is frequently detected in pathologically confirmed AD brains in up to 57% of AD cases [10, 115–123], where it has been associated with worse brain atrophy and greater memory loss in AD patients [116]. TDP-43 species have been shown to colocalize with senile plaques and neurofibrillary tangles, with experimental evidence suggesting a direct interaction between TDP-43 and Aβ or tau [122, 124–128]. Furthermore, TDP-43 pathology in AD is associated with the severity of AD pathology, including higher Braak neurofibrillary tangle stages and Thal amyloid phases [129]. Additionally, TDP-43 NCIs in AD cases exhibit a variety of TDP-43 species with distinct patterns in terms of TDP-43 phosphorylation sites and the presence or absence of non-phosphorylated, N-terminal and C-terminal epitopes [130]. Altogether, it suggests that TDP-43 pathology could play a role in AD progression or be secondary to reactive changes that occur in advanced AD (Fig 3).

#### Clinical significance of TDP-43 in AD

TDP-43 has been reported to influence the clinical features of dementia, including cognitive deficits and the likelihood of dementia. Josephs and coworkers sought to determine the frequency of TDP-43 pathology across AD subtypes and its effects on cognition [119]. They found that deposition of TDP-43 was frequent in limbic predominant (67%) and typical AD subtypes (59%), but less frequent in the hippocampal sparing subtype (21%) [119]. Although the frequency of TDP-43 deposition in AD varies by pathological subtype, the observed effects of TDP-43 on clinical features, such as exacerbating cognitive decline, were consistent across pathological subtypes [119]. Another study investigated TDP-43, mixed pathologies, and clinical AD type dementia in the Religious Orders Study and the Rush Memory and Aging Project (ROSMAP) cohort with 946 old-age adults (89.3 ± 6.5 years) [115]. TDP-43 pathology was present in 52% of the participants; 65% in individuals with Alzheimer’s-type dementia and 44% in cognitively normal individuals. Additionally, coexistence of both TDP-43 and AD pathology was more common in those with Alzheimer’s-type dementia (54%) than those without dementia (25%) [115]. After using a logistic regression model and accounting for age, sex, and education, the investigators discovered that not only mixed AD and TDP-43 pathology, but also TDP-43 pathology, alone, was associated with Alzheimer’s-type dementia with an odds ratio of 6.73 and 1.51, respectively [115]. Similarly, McAleese and coworkers investigated the frequency of TDP-43 pathology in 119 individuals with autopsy-confirmed AD, dementia with Lewy bodies (DLB), mixed AD/DLB, and non-demented elderly controls [120]. TDP-43 pathology was present in all groups but was the highest in AD (73.9%) and mixed AD/DLB (52.6%) groups.

Overall, these results suggest that TDP-43 pathology is common in AD, especially in the limbic predominant subtype. These results also suggest TDP-43 pathology is a risk factor for developing dementia of the Alzheimer type independent of pathological subtypes, and TDP-43 pathology increases the rate of hippocampal atrophy in AD.

#### Progression pattern of TDP-43 pathology in AD

**Fig. 4:**
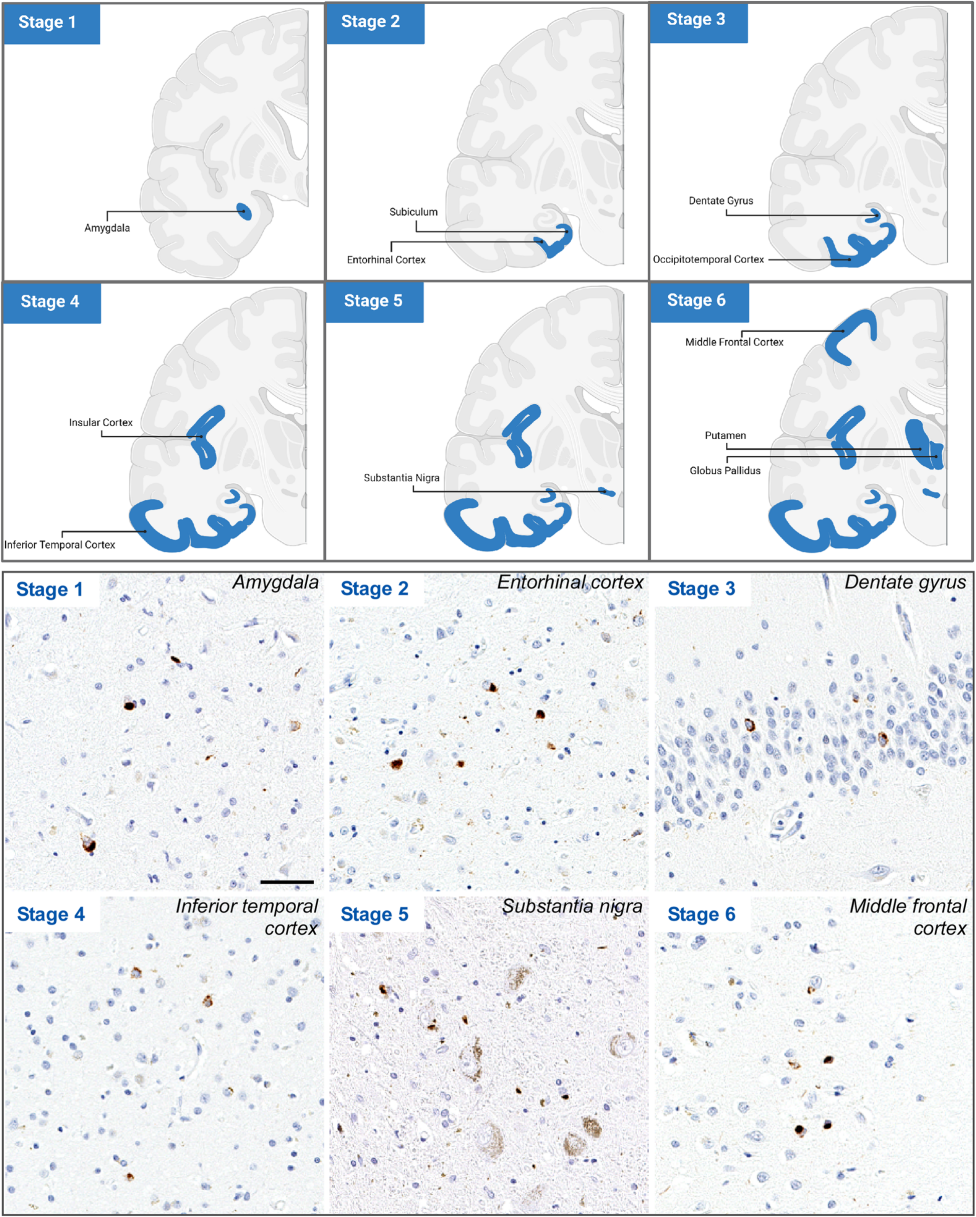
Distribution pattern of TDP-43 pathology in AD. (Upper panel) Illustration of TDP-43 stage in AD. An anterior coronal section depicts TDP-43 pathology progression from the amygdala (Stage 1), into the subiculum and entorhinal cortex (Stage 2), and then leads into the occipitotemporal cortex and dentate gyrus (Stage 3) of the hippocampus, followed by the insular cortex and the inferior temporal cortex (Stage 4). After progressing into the substantia nigra (Stage 5), the pathology reaches its final stage at the basal ganglia (putamen and globus pallidus) and middle frontal cortex (Stage 6). (Lower panel) Immunohistochemistry with an anti-phosphorylated-TDP-43 antibody shows representative images of TDP-43 pathology in different brain regions of each stage. Stage 1, amygdala; Stage 2, entorhinal cortex; Stage 3, dentate gyrus; Stage 4, inferior temporal gyrus; Stage 5, substantia nigra; Stage 6, middle frontal gyrus. Scale bar = 50 μm

Interestingly, TDP-43 presenting as a secondary comorbid pathology in AD follows its own distinct pathological distribution pattern compared to that of ALS and FTLD. Josephs et al proposed that the progression of TDP-43 pathology in AD occurs in six stages, with stage 1 being characterized by TDP-43 pathology present within the amygdala (Fig 4) [117]. Progression into the entorhinal cortex and subiculum of the hippocampus defines stage 2, while stage 3 involves the hippocampal dentate gyrus and occipitotemporal cortex. In a subset of cases, the hippocampus has neuronal loss and gliosis consistent with hippocampal sclerosis [118, 123], but in other cases TDP-43 pathology is associated with Alzheimer type lesions, in particular neurofibrillary tangles [123]. The phenomenon of TDP-43 colocalization in neurons with neurofibrillary tangles has been termed Type β [131], to distinguish it from genuine NCI in Type B cellular pathology. As the pathology progresses into stage 4, the insular cortex, ventral striatum, basal forebrain, and inferior temporal cortex become affected. In stage 5, TDP-43 pathology now involves the brainstem nuclei, including the substantia nigra, inferior olivary nucleus, and midbrain tectum. The final stage, stage 6, is associated with involvement of basal ganglia and middle frontal cortex [117]. The TDP-43 stage was not affected by the age at onset, nor the time from onset to death in these AD patients [117]. This staging scheme is supported by assessment of clinical behavior, pathological characteristics, neuroimaging, and genetics; however, the underlying mechanisms driving distribution of TDP-43 in AD is unclear.

#### TDP-43 and Aβ

*In vitro* and *in vivo* data have indicated that pathologic processes leading to AD and those leading to TDP-43 aggregation may influence one another. One study found that full length recombinant TDP-43 can form stable and spherical oligomers that can be recognized and bound by A11, an anti-amyloid oligomer specific antibody [92]. TDP-43 oligomers found in AD and FTLD brains [92, 127] are toxic to neurons both *in vitro* and *in vivo* through mechanisms that include reducing the DNA binding capacity of TDP-43, suggesting that oligomerization of TDP-43 may lead to gain of toxic function, as well as loss of physiological function [92]. The investigators also noted that soluble Aβ is converted to Aβ oligomers in the presence of TDP-43 oligomers due to their ability to cross-seed [92, 132]. This suggests that TDP-43 and Aβ have structurally similar domains that could contribute to the formation of Aβ-TDP-43 complexes. The frequent detection of TDP-43 positive inclusion bodies in AD could be due in part to this potential cross-seeding capacity of Aβ with TDP-43 [132]. Interestingly, full length TDP-43, as well as truncated N-terminal and C-terminal variants, were found to reduce Aβ fibrillization in a dose-dependent manner at oligomeric and other pre-fibril stages [92, 125]. Analogous to the most significant deficits seen in humans with AD and TDP-43 pathologies, mice with recombinant TDP-43 oligomers injected into the hippocampus had exacerbation of neuroinflammation and memory deficits [125].

A study investigating the relationship between TDP-43 and AD found that late stage AD patients have increased pathological cortical TDP-43 [122], which is consistent with the finding that TDP-43 pathology is associated with severe AD pathology [129]. Similar to late-stage AD, the investigators also noted an increase in TDP-43 pathology after Aβ (1-42) expressing lentiviral injections into the cortices of rats, as well as co-localization of intracellular Aβ with TDP-43, and association between phospho-TDP-43 and Aβ [122]. These data suggest a direct relationship between pathological TDP-43 and expression of Aβ in cells [122].

Another study revealed that the overexpression of TDP-43 in the cortex and hippocampus of an APP/PS1 mouse model (carrying mutant *APP* and *PSEN1* genes) resulted in a decrease in Aβ plaque burden [124]. In this TDP-43 overexpression model there was also increased formation of TDP-43 oligomers [124]. In addition, there were increased levels of the amyloid precursor protein (APP) in the lysosomes, which might be the explanation for reduced Aβ plaques rather than inhibition of amyloid fibrilization by direct interaction with Aβ and extracellular TDP-43.

In another model system, there was increased neurodegeneration in the hippocampus of an APP/PS1 mouse model with conditional TDP-43 knockout in the forebrain [133], suggesting that TDP-43 depletion may contribute to neurodegeneration. Perhaps loss of TDP-43 function due to pathological modifications and mislocalization in a background of AD pathology may function in a similar way to TDP-43 depletion, possibly exacerbating neurodegeneration similar to results observed in AD [116]. Interestingly, the APP/PS1 TDP-43 knockout mice had a decrease in Aβ burden but increased oligomeric Aβ levels [133], suggesting that both overexpression and depletion of TDP-43 result in similar Aβ outcomes. Similarly, microglial-specific inducible conditional TDP-43 knockout in an APP mouse line was found to increase phagocytic activity of microglia, which resulted in increased amyloid clearance and reduction in Aβ plaque burden [134]. Additionally, microglial-specific TDP-43 depletion induced synaptic loss, even in the absence of amyloid, which may contribute to downstream neurodegeneration possibly due to synaptic pruning by overactive microglia [134]. These data suggest that microglial phagocytic activity, and thus Aβ clearance, may be at least in part regulated through TDP-43.

#### TDP-43 and Tau

Cytoplasmic inclusions in FTLD are typically immunoreactive for either Tau or TDP-43, respectively, thus the clarification of two subtypes FTLD-Tau and FTLD-TDP [135]. However, there has been studies that investigated the relationship between tau and TDP-43 outside the context of FTLD. For example, an *in vitro* study revealed that tau oligomer treatment increased nuclear levels of both phosphorylated and non-phosphorylated TDP-43 monomers in a dose-dependent manner [127]. Additionally, as the concentration of tau oligomers increased, the levels of phosphorylated TDP-43 oligomers in the cytoplasm increased as well, resulting in accumulations of phosphorylated TDP-43 oligomers that were also immunoreactive for tau oligomers [127], suggesting that the presence of tau oligomers induces the mislocalization and polymerization of TDP-43 species into oligomers and aggregates, and that tau oligomers may be able to cross-seed with TDP-43. Furthermore, TDP-43 oligomers were found to co-localize with tau and Aβ in AD and FTLD post-mortem brains [127].

The previously discussed APP/PS1 mouse model with TDP-43 overexpression was associated with increased pathological tau, which suggests that TDP-43 could play a role in neurofibrillary tangle development [124]. Furthermore, phosphorylated tau was present within mouse neuronal extensions in APP/PS1 transgenic mice with TDP-43 overexpression. In addition, colocalization of TDP-43 and phosphorylated tau has been detected in AD brains, with distinct tau and TDP-43 filaments within the same neuron [123, 136]. These data suggest that depending on the context, TDP-43 and tau may influence one another’s pathological progression; TDP-43 can promote pathological tau accumulation, or vice versa. However, an inverse association between TDP-43 and tau within post-mortem AD brains was also reported, possibly due to the negative regulation of tau transcripts by TDP-43 [18]. Therefore, additional studies are required to elucidate the relationship between TDP-43 and tau in AD development.

#### TDP-43 and *APOE* in AD

Apolipoprotein E (apoE), a glycoprotein present within the central nervous system and periphery, is an important lipid transporter, especially for cholesterol [137]. The human *APOE* gene has three alleles: *APOE2*, *APOE3*, and *APOE4*, with *APOE2* being associated with the reduced risk for late-onset AD, while *APOE4* is a major risk factor for late-onset AD [137–139]. ApoE has been well-known to influence Aβ pathology, as well as other neurodegenerative disease pathologies, including α-synuclein, in an isoform-dependent manner [118, 140–144]. Associations between *APOE4* and TDP-43 pathology have also been reported [116, 118, 145]. A case study suggested that apoE and TDP-43 can form complexes based on co-immunoprecipitation data, and that *APOE* genotype can affect the severity of the complex burden with the *APOE4/4* individual suffering from a higher burden compared to *APOE3/3* [146]. Using a cohort from Mayo Clinic’s brain bank, Josephs and coworkers determined that pathologically confirmed AD patients with TDP-43 co-pathology were also more likely to carry the *APOE4* allele when compared to TDP-43 negative AD cases [116]. Additionally, these individual’s scores on multiple cognitive impairment tests were decreased and cognitive impairment was more likely to present itself before death [116]. Similarly, another study based upon the ROSMAP cohort has reported that the stage and burden of TDP-43 pathology are positively correlated with the number of *APOE4* alleles, even after controlling for amyloid, tau, and Lewy body pathologies [118]. Wennberg and coworkers analyzed a cohort of 751 pathologically confirmed AD cases for TDP-43 status, *APOE* genotype, tau neurofibrillary tangle stage, and Aβ status and found a direct association between *APOE4* and TDP-43; the association was mediated by Aβ and tau [145]. Overall, these data suggest that *APOE4* increases TDP-43 burden and likely increases the risk of TDP-43 pathology in AD by processes linked to Alzheimer type pathology and also processes independent of Aβ, thus contributing to detrimental effects of *APOE4* on cognition later in life.

### TDP-43 pathology in aging and hippocampal sclerosis (HS) of the elderly

Age-dependent demethylation of the *TARDBP* 3′ untranslated region has been reported to increase *TARDBP* mRNA expression in the motor cortex in ALS [147]. Besides ALS, aging is considered a risk factor for developing TDP-43 pathology even in neurologically normal individuals [148–150]. From 286 consecutive autopsy brains, Uchino and coworkers reported that 40% of control elderly individuals (78.5 ± 9.7 years) with minimal senile plaques had TDP-43 pathology [151]. Additionally, TDP-43-positive individuals were reported to be significantly older than those without TDP-43 pathology from a study investigating TDP-43 in the anterior temporal pole cortex [152]. These data suggest that TDP-43 pathology in the anterior temporal pole cortex is an important early neocortical stage of TDP-43 progression in aging and AD while extension of TDP-43 pathology to the midfrontal cortex is a late stage associated with more severe and global cognitive impairment [152]. Similarly, a study exploring age-related interneuron degeneration discovered that aged TDP-43 transgenic mice suffered from a significantly higher amount of TDP-43 positive inclusions than did non-transgenic aged mice as well as worse degeneration [153].

Hippocampal sclerosis (HS) increases in frequency with age and is a distinct process from AD, even though they both are associated with an amnestic clinical syndrome [154]. About 10-25% of individuals over the age of 85 are affected by HS-aging with the pathological feature of TDP-43 pathology in the hippocampus [150]. Neuronal loss in HS overlaps with that seen in epilepsy and hypoxic-ischemia, but the latter are not associated with TDP-43 pathology [155]. The discovery of TDP-43 pathology in HS of the elderly was the first evidence that this was a unique disease process that is associated with advanced age. Common genetic variants in *GRN* and *TMEM106B* are risk factors for FTLD [156, 157] and subsequent studies have also shown that they are risk factors for HS of the elderly [158, 159], linking this old age pathology to a similar disease process associated with FTLD. The *GRN* and *TMEM106B* genetic associations have also been observed in HS in the setting to Lewy body dementia, most of whom have at least some co-existing Alzheimer type pathology [160].

Given the fact that HS can be associated with degenerative, toxic, and selective hippocampal neuronal loss associated with anoxic-ischemic injury or epilepsy, the term HS has fallen out of favor. An international group of experts proposed a new name for TDP-43 pathology in the elderly, often associated with HS, “limbic-predominant age-related TDP-43 encephalopathy” (LATE) [149]. LATE neuropathological change (LATE-NC) is the term to refer to the pathology to distinguish it from the clinical syndrome, LATE, which remains to be defined, but is clearly associated with at least an amnestic syndrome. LATE-NC is characterized by TDP-43 neuronal and glial inclusions, with or without neuronal loss. TDP-43 pathology in LATE is concentrated in the limbic regions, including the amygdala, hippocampus, and anterior cingulate gyrus. According to a simplified staging scheme of LATE-NC, TDP-43 pathology initially forms in the amygdala (stage 1) and then extends to the hippocampus (stage 2) and the middle frontal gyrus (stage 3). Although it remains controversial [161], LATE can be differentiated from FTLD-TDP based on its epidemiology and severity of cortical TDP-43 pathology. LATE usually affects much older adults (present in 20-50% of individuals past 80 years old) than FTLD-TDP [149, 162]. TDP-43 pathology in the middle frontal gyrus in LATE-NC stage 3 is less severe than that of FTLD-TDP [163]. LATE is commonly found with co-pathologies including Aβ and tau [149]. Indeed, AD and LATE are often comorbid processes. LATE has been linked with robust disease-specific cognitive impairment, and it is one of the common age-related diseases that can imitate AD [149]. In the ROSMAP cohort, 15-20 percent of clinically diagnosed AD dementia patients at 80 years of age or older are associated with LATE [149].

### TDP-43 and other neurodegenerative disorders

TDP-43 has been reported as a co-pathology in other neurodegenerative disorders besides AD, including Huntington’s disease, progressive supranuclear palsy (PSP), corticobasal degeneration (CBD), argyrophilic grain disease, DLB, and multiple system atrophy (MSA) [10, 120, 126, 128, 164–170]. In most cases, phosphorylated or truncated TDP-43 is a component of the cytoplasmic inclusions in these disorders, occasionally co-localizing with the primary pathology [120, 125–127]. The prevalence of the co-pathology depends on the primary pathology. For instance, over 57% of AD patients or 45% of CBD patients had TDP-43 pathology, while less than 6% of PSP or MSA patients had TDP-43 pathology [10, 116, 164, 168, 169].

An i*n vivo* study using transgenic mice expressing human TDP-43 mutants found that administration of an autophagy-inducing drug could ameliorate TDP-43 pathology in the brain and spinal cord of the transgenic animals [171]. Given the fact that tau and α-synuclein pathologies also implicate disruption of autophagic pathways [172–174], developing active pharmacological agents to enhance autophagy flux may alleviate intracellular aggregation-prone proteins. Due to the ubiquitous nature of TDP-43 expression, it may not be a viable therapeutic approach to target TDP-43 in a generalized manner; however, strategies to modify the TDP-43 toxicity and to reduce TDP-43 aggregation may not only benefit FTLD and ALS patients [175], but also be relevant to more common age-related neurodegenerative disorders such as AD, Lewy body dementia, and LATE.

## Conclusions

Significant efforts in the past decade have been placed in finding and testing new treatment methods for AD in hopes to prevent or cure this devastating disease. TDP-43 pathology, commonly found in AD brains, has been shown to influence AD pathology and neurodegeneration, whether it be decreasing senile plaque load through overexpression, or increasing amyloid oligomers and synapse loss through depletion. It also shares an important genetic risk factor with AD, the *APOE4* gene. The mere presence of TDP-43 pathology increases the likelihood of developing Alzheimer-type dementia. These findings provide strong evidence for TDP-43 being an integral part of multiple neurodegenerative conditions, emphasizing the need to better understand the mechanisms of TDP-43 pathogenesis in AD and other age-related disorders.

## Data Availability

Not applicable.

## Abbreviations

**Aβ**: Amyloid-β
**AD**: Alzheimer’s disease
**ALS**: Amyotrophic lateral sclerosis
***APOE***: Apolipoprotein E
**CBD**: Corticobasal degeneration
**CTF**: C-terminal fragment
**DG**: Dentate gyrus
**DLB**: Dementia with Lewy bodies
**DN**: Dystrophic neurite
**FTD**: Frontotemporal dementia
**bvFTD**: Behavioral variant frontotemporal dementia
**FTLD**: Frontotemporal lobar degeneration
**GCI**: Glia cytoplasmic inclusion
**GRD**: Glycine rich domain
**hnRNP**: Heterogeneous nuclear ribonuclear protein
**HS**: Hippocampal sclerosis
**LATE**: Limbic-predominant age related TDP-43 encephalopathy
**LATE-NC**: LATE neuropathological change
**MND**: Motor neuron disease
**MSA**: Multiple system atrophy
**NCI**: Neuronal cytoplasmic inclusion
**NES**: Nuclear export signal
**NII**: Neuronal intranuclear inclusion
**NLS**: Nuclear localization sequence
**PNFA**: Progressive non-fluent aphasia
**PSP**: Progressive supranuclear palsy
**RBD**: RNA binding domain
**ROSMAP**: Religious Orders Study and Rush Memory and Aging Project
**SD**: Semantic dementia
**SOD1**: Superoxide dismutase
**TDP-43**: Transactive response DNA binding protein of 43 kDa
